# Efficient Implementation of Equation-of-Motion Coupled-Cluster Singles and Doubles Method with the Density-Fitting Approximation: An Enhanced Algorithm for the Particle–Particle Ladder Term

**DOI:** 10.1021/acs.jctc.1c01000

**Published:** 2022-02-02

**Authors:** Aslı Ünal, Uğur Bozkaya

**Affiliations:** † Graduate School of Science and Engineering, Hacettepe University, Ankara 06800, Turkey; ‡ Department of Chemistry, Hacettepe University, Ankara 06800, Turkey

## Abstract

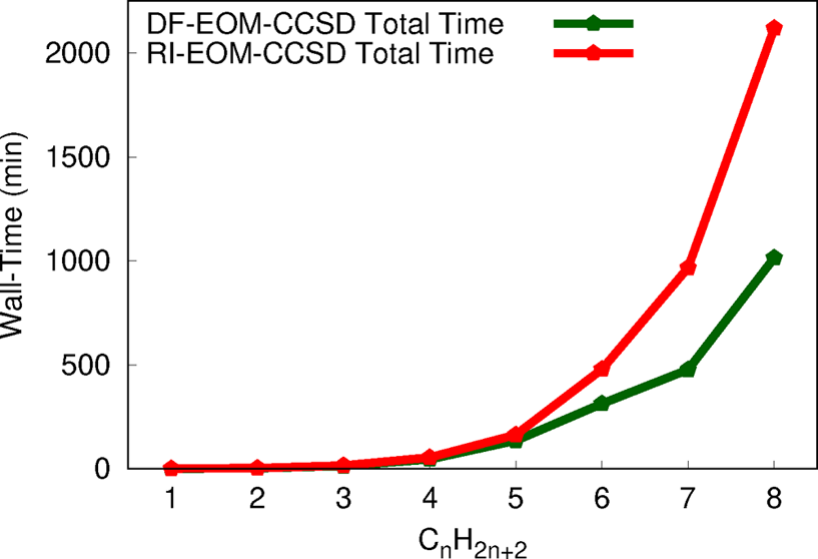

An efficient implementation of the density-fitted equation-of-motion coupled-cluster singles and doubles (DF-EOM-CCSD) method is presented with an enhanced algorithm for the particle–particle ladder (PPL) term, which is the most expensive part of EOM-CCSD computations. To further improve the evaluation of the PPL term, a hybrid density-fitting/Cholesky decomposition (DF/CD) algorithm is also introduced. In the hybrid DF/CD approach, four virtual index integrals are constructed on-the-fly from the DF factors; then, their partial Cholesky decomposition is simultaneously performed. The computational cost of the DF-EOM-CCSD method for excitation energies is compared with that of the resolution of the identity EOM-CCSD (RI-EOM-CCSD) (from the Q-chem 5.3 package). Our results demonstrate that DF-EOM-CCSD excitation energies are significantly accelerated compared to RI-EOM-CCSD. There is more than a 2-fold reduction for the C_8_H_18_ molecule in the cc-pVTZ basis set with the restricted Hartree-Fock (RHF) reference. This cost savings results from the efficient evaluation of the PPL term. In the RHF based DF-EOM-CCSD method, the number of flops (NOF) is 1/4*O*^2^*V*^4^, while that of RI-EOM-CCSD was reported (Epifanovsky et al. J. Chem. Phys.2013, 139, 1341052411655010.1063/1.4820484) to be 5/8*O*^2^*V*^4^ for the PPL contraction term. Further, the NOF of *VVVV*-type integral transformation is 1/2*V*^4^*N*_*aux*_ in our case, while it appears to be *V*^4^*N*_*aux*_ for RI-EOM-CCSD. Hence, our implementation is 2.5 and 2.0 times more efficient compared to RI-EOM-CCSD for these expensive terms. For the unrestricted Hartree-Fock (UHF) reference, our implementation maintains its enhanced performance and provides a 1.8-fold reduction in the computational time compared to RI-EOM-CCSD for the C_7_H_16_ molecule. Our results indicate that our DF-EOM-CCSD implementation is 1.7 and 1.4 times more efficient compared with RI-EOM-CCSD for average computational cost per EOM-CCSD iteration. Moreover, our results show that the new hybrid DF/CD approach improves upon the DF algorithm, especially for large molecular systems. Overall, we conclude that the new hybrid DF/CD PPL algorithm is very promising for large-sized chemical systems.

## 1 Introduction

It is well-known that coupled-cluster (CC) methods provide accurate results for molecular properties for most chemical systems near equilibrium geometries.^1−13^ For example, the coupled-cluster singles and doubles (CCSD) method^14^ provides quite accurate results for most molecular systems at equilibrium geometries. The addition of a perturbative triples excitations correction [CCSD(T)]^10,11,15^ further enhances CCSD and yields very accurate results for a broad range of molecular systems.^12,16−25^ However, high computational costs of common CC methods, such as *O*(*N*^6^) and *O*(*N*^7^) for CCSD and CCSD(T) (where *N* is the number of basis functions), limits their applications to relatively small-sized chemical systems.

Accurate computations of excitation energies (EEs) is one of the most challenging problems in modern quantum chemistry. Equation-of-motion CC (EOM-CC) methods provide accurate results for excited-state properties for a broad range of chemical systems.^26−44^ The accuracy of the EOM approach based on the CCSD model (EOM-CCSD) has been reported to be 0.1–0.2 eV.^28,31^ However, as in the case of the ground-state CC methods, the computational cost and disk/memory requirements for the EOM-CC methods scale steeply with the system size.

Tensor decomposition techniques for electron repulsion integrals (ERIs) have been of significant interest in modern computational chemistry.^45−70^ Density fitting (DF) is one of the most popular ERI decomposition techniques.^45−52,59−70^ In the DF approach, the ERI tensor of rank-4 is expanded in terms of rank-3 tensors. Another common ERI factorization approach is the partial Cholesky decomposition (CD).^55−60,63,64^ The DF and CD techniques are very useful to reduce the cost of integral transformations and the storage requirements for the ERI tensor.

In this research, a new implementation of the density-fitted EOM-CCSD method is presented with an enhanced algorithm for the particle–particle ladder (PPL) term, which is the most expensive term. The equations presented have been implemented in a new computer code by the present authors and added to the MacroQC package.^71^ The computational time of our DF-EOM-CCSD implementation is compared with that of the Q-chem 5.3 software.^72^ The DF-EOM-CCSD method is applied to a test set for excitation energies.

## 2 CCSD Energy and Amplitude Equations

At first, we would like to note that all equations reported in this study are in the spin–orbital formalism. The spin-free version of our equations for the restricted closed-shell systems are provided in the Supporting Information. The unrestricted version of the formulas can be readily obtained from the spin–orbital equations.

The correlation energy for the CCSD method can be expressed as follows
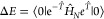
1where *Ĥ*_*N*_ is the normal-ordered Hamiltonian operator,^4,73^ |0⟩ is the Hartree–Fock (HF) determinant, and *T̂* is the sum of single- and double-excitation operators *T̂* = *T̂*_1_ + *T̂*_2_:
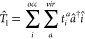
2
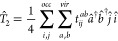
3where *â*^†^ and *î* are the creation and annihilation operators and *t*_*i*_^*a*^ and *t*_*ij*_^*ab*^ are the single and double excitation amplitudes, respectively.

*t*_*i*_^*a*^ and *t*_*ij*_^*ab*^ can be obtained from the following equations:
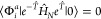
4
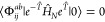
5where Φ_*i*_^*a*^ and Φ_*ij*_^*ab*^ are singly and doubly excited Slater determinants, respectively. For explicit equations of our CCSD implementation, one may refer to our previous studies.^65−67^

## 3 The EOM-CCSD Model

In the EOM-CCSD framework, the target excited-state wave functions are written as follows:
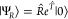
6
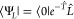
7where *R̂* and *L̂* are linear excitation and de-excitation operators, respectively. For EOM-CCSD, *R̂* = *R̂*_1_ + *R̂*_2_:
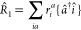
8
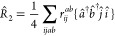
9where *r*_*i*_^*a*^ and *r*_*ij*_^*ab*^ are the single and double excitation amplitudes, respectively, and the notation {*â*^†^...*î*} denotes a string of normal-ordered operators with respect to the Fermi vacuum.

For the ground state, we have the following Schrödinger equation:
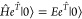
10Hence, by multiplying eq 10 by *e*^–*T̂*^, we obtain
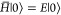
11where *H̅* = *e*^–*T̂*^*Ĥe*^*T̂*^.

Further, the normal ordered *H̅* can be written as follows:
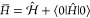
12Hence, we define:
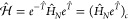
13where subscript *c* means that only connected diagrams should be included. Therefore, we may rewrite eq 11 as follows
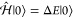
14where Δ*E* is the ground-state CC correlation energy.

The excited-state eigenvalue equation can be written as follows:
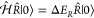
15where Δ*E*_*R*_ is the excited-state CC correlation energy. The excitation energy can be written as
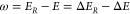
16

After performing some algebra, we obtain the EOM-CCSD equation as follows:
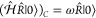
17Equation 17 is equivalent to the following matrix eigenvalue equation for CCSD:
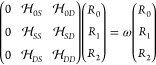
18However, we solve eq 18 iteratively with the Davidson algorithm.^74−77^ Hence, we need to introduce the so-called **σ** vector as follows:
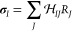
19where *I*, *J* = 0, *S*, *D*.

### 3.1 DF-EOM-CCSD Intermediates

The DF-CCSD intermediates that appear in the DF-EOM-CCSD equations are given in Appendix A.

#### 3.1.1 DF-EOM-CCSD 3-Index Intermediates

1- and 3-index intermediates that were used for EOM-CCSD are defined as follows:
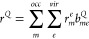
20
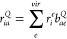
21
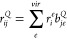
22
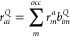
23
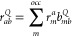
24
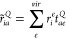
25
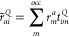
26
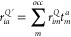
27
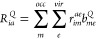
28where *Q* runs over auxiliary basis functions and the *b*_*pq*_^*Q*^ terms are the molecular orbital (MO) basis DF factors, which are defined in our previous studies.^65−67^

#### 3.1.2 4-Index Intermediates

4-index intermediates are defined as follows:
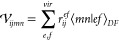
29
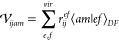
30
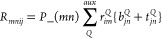
31
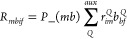
32
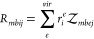
33

#### 3.1.3 2-Index Intermediates

2-index intermediates are defined as follows:
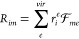
34
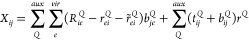
35
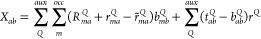
36

### 3.2 DF-EOM-CCSD σ Equations

The DF-EOM-CCSD σ_0_ equation can be written as
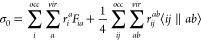
37

With the DF approximation, the EOM-CCSD σ_*i*_^*a*^ equation can be written as
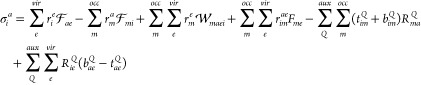
38

With the DF approximation, the EOM-CCSD σ_*ij*_^*ab*^ equation can be written as
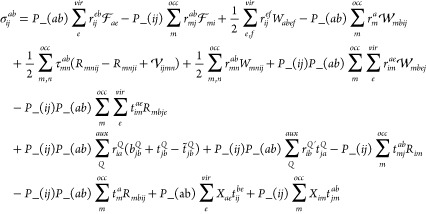
39

### 3.3 PPL Algorithm with the DF Approach

The most expensive terms of the *T*_2_ and σ_2_ amplitude equations are the PPL terms. Our PPL algorithm for the σ_2_ tensor originated from the PPL algorithm used for the *T*_2_ amplitude equation in our 2016 study.^65^ Here, we employ the same algorithm to σ_2_ amplitudes. For example, for the closed-shell case, the PPL term can be written as
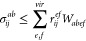
40Following the previous studies of Saebø and Pulay^78^ and Scuseria at al.^79^ and our previous DF-CCSD studies,^65,66^ we employ the following algorithm for the evaluation of σ-PPL:
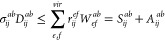
41where *S* is the symmetric component, while *A* is the antisymmetric component. Now let us define
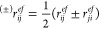
42
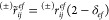
43
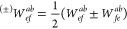
44
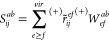
45
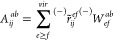
46where *S* and *A* have the following symmetry properties.
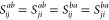
47
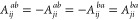
48Hence, we can always keep *i* ≥ *j* and *a* ≥ *b*.
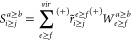
49
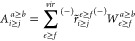
50

The pseudo code for the σ-PPL algorithm is
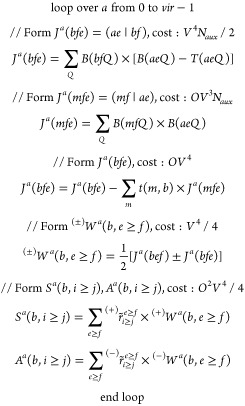
51With this algorithm, the cost of PPL is 1/4*O*^2^*V*^4^ + 1/2*V*^4^*N*_*aux*_ + *OV*^3^*N*_*aux*_ + *OV*^4^ + 1/4*V*^4^. The most expensive term is 1/2*V*^4^*N*_*aux*_. Finally, we note that our DF-EOM-CCSD code has a shared-memory parallelism feature through threaded BLAS calls as well as OpenMP parallelization of all tensor manipulations.

#### 3.3.1 DF/CD Hybrid PPL Algorithm

In the common CD approach for ERIs, the CD factors are generated from the AO basis ERI tensor (μν|λσ), and the number of CD factors is generally higher than that of DF factors. Hence, it does not seem to speed up our DF algorithm. However, we have observed that, for the large molecules, the CD technique can be beneficial to take advantage of the sparsity of the ERI tensor if it is applied to the MO basis ERIs generated from the DF integrals. More specifically, if we perform the Cholesky decomposition of the (*ab*|*cd*)-type integrals, we may get a reduced number of auxiliary basis functions, which is especially true for large molecular systems. Hence, in our DF/CD hybrid approach, we build the (*ab*|*cd*)-type integrals from the DF factors, on-the-fly, and perform Cholesky decomposition simultaneously.

## 4 Results and Discussion

Results from the DF-EOM-CCSD and RI-EOM-CCSD^80^ methods were obtained for a set of alkanes to compare the computational cost for the excitation energy computations. For the alkanes set, Dunning’s correlation-consistent polarized valence triple-ζ basis set (aug-cc-pVTZ) was used with the frozen core approximation.^81,82^ For the aug-cc-pVTZ basis sets, aug-cc-pVTZ-JKFIT^50^ and aug-cc-pVTZ-RI^83^ auxiliary basis set pairs were employed for reference and correlation energies, respectively. Additionally, the DF-EOM-CCSD, resolution of the identity EOM-CCSD (RI-EOM-CCSD),^80^ and EOM-CCSD(fT)^84^ methods were applied to a set of molecules to compare the excitation energies.

### 4.1 Efficiency of DF-EOM-CCSD

**Figure 1:**
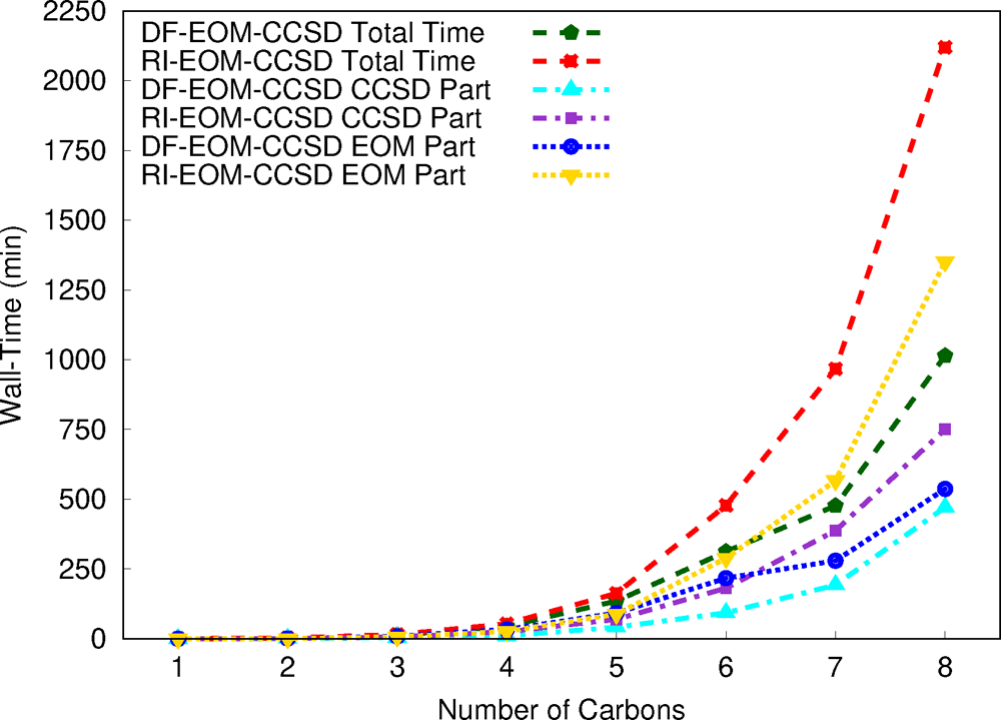
Total, CCSD, and EOM wall times (in min) for computations of excitation energies for the C_*n*_H_2*n*+2_ (*n* = 1–8) set from the RI-EOM-CCSD (from Q-Chem(72)) and DF-EOM-CCSD methods with the cc-pVTZ basis set. The RHF reference is used for these computations. All computations were performed for a single root with 10^–7^ energy and EOM eigenvalue convergence tolerances on a single node (1 core) Intel(R) Xeon(R) CPU E5-2620 v4 @ 2.10 GHz computer (memory ∼ 64 GB).

**Figure 2:**
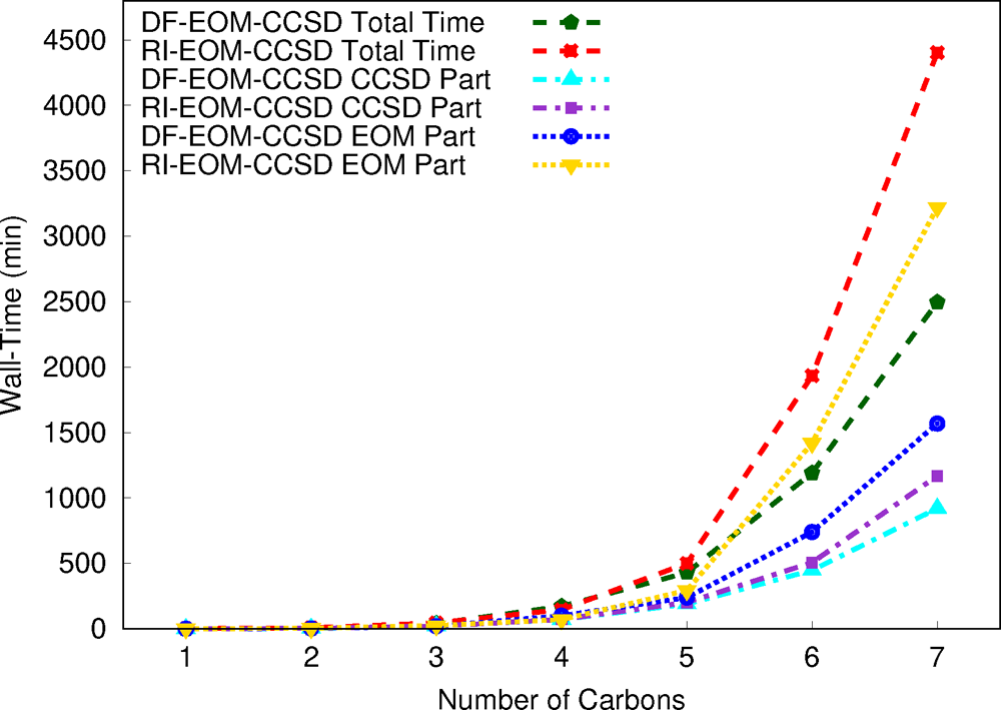
Total, CCSD, and EOM wall time (in min) for computations of excitation energies for the C_*n*_H_2*n*+2_ (*n* = 1–7) set from the RI-EOM-CCSD (from Q-Chem(72)) and DF-EOM-CCSD methods with the cc-pVTZ basis set. The UHF reference is used for these computations. All computations were performed for a single root with 10^–7^ energy and EOM eigenvalue convergence tolerances on a single node (1 core) Intel(R) Xeon(R) CPU E5-2620 v4 @ 2.10 GHz computer (memory ∼ 64 GB).

A set of alkanes is considered to assess the efficiency of the RI-EOM-CCSD and DF-EOM-CCSD methods. The RI-EOM-CCSD computations were performed with the Q-chem 5.3 package.^72^ The computational time for the RI-EOM-CCSD and DF-EOM-CCSD methods are presented graphically in Figures 1 and 2 for restricted and unrestricted Hartree-Fock (RHF and UHF) references, respectively. Timing computations were carried out for a single root with a 10^–7^ energy and 10^–7^ EOM eigenvalue convergence tolerances on a single node (1 core) Intel(R) Xeon(R) CPU E5-2620 v4 @ 2.10 GHz computer (memory ∼ 64 GB). For the RI-CCSD code of Q-chem 5.3, MEM_TOTAL 64000, MEM_STATIC 2000, and CC_MEMORY 51200 options are used. We start our assessment with the RHF versions of the RI-EOM-CCSD and DF-EOM-CCSD implementations. The DF-EOM-CCSD method significantly reduces the computational cost compared to RI-EOM-CCSD, and there is more than a 2-fold reduction in the computational time for DF-EOM-CCSD for the largest member (C_8_H_18_) of the alkanes set. For the C_8_H_18_ molecule, the CCSD times are 750 and 471 min for RI-EOM-CCSD and DF-EOM-CCSD, respectively; there is a 1.6-fold reduction in the computational time for DF-EOM-CCSD. Further, for the C_8_H_18_ molecule, the EOM times are 1351 min (RI-EOM-CCSD) and 537 min (DF-EOM-CCSD); hence, there is a 2.5-fold reduction in the computational time for DF-EOM-CCSD.

The number of iterations for the CCSD part are 11 (DF-EOM-CCSD) and 12 (RI-EOM-CCSD). The average computational time per CCSD iteration (*t*_*ccsd*_/*n*_*iter*_) for C_8_H_18_ is 42.8 min (DF-EOM-CCSD) and 62.5 min (RI-EOM-CCSD). Hence, our new DF-EOM-CCSD implementation is 1.5 times faster than the RI-EOM-CCSD code for average computational cost per CCSD iteration. Similarly, the number of Davidson iterations for the EOM part is 12 (DF-EOM-CCSD) and 18 (RI-EOM-CCSD). The average computational time per a Davidson iteration (*t*_*eom*_/*n*_*iter*_) for C_8_H_18_ is 44.8 min (DF-EOM-CCSD) and 75.1 min (RI-EOM-CCSD). Hence, our new DF-EOM-CCSD implementation is 1.7 times faster than the RI-EOM-CCSD code for average computational cost per EOM-CCSD iteration.

The efficiency of our DF-EOM-CCSD method compared to that of RI-EOM-CCSD is attributed to the our more efficient PPL algorithm. For the closed-shell case, the number of flops (NOF) for our DF-CCSD method^65,66^ is 1/4*O*^2^*V*^4^ + 2*O*^3^*V*^3^ + 1/4*O*^4^*V*^2^, while that of RI-CCSD^80^ was reported to be 5/8*O*^2^*V*^4^ + 4*O*^3^*V*^3^ + 27/8*O*^4^*V*^2^. When one compares the cost of implementation, our DF-CCSD implementation^65,66^ is 2.5 times more efficient than that of RI-CCSD^80^ for the PPL contraction term. Further, our implementation is 2 times more efficient compared to that of RI-CCSD for the particle-hole ladder (PHL)terms. Moreover, the cost of *VVVV*-type integral transformation, on-the-fly of course, is 1/2*V*^4^*N*_*aux*_ in our case, while it appears to be *V*^4^*N*_*aux*_ for RI-CCSD.^80^ In fact, the most expensive term is this integral transformation step for large-scale computations with optimized auxiliary basis sets. Hence, our algorithm appears to be 2 times more efficient for this term. Basically, we follow the same algorithm for the PPL term of EOM; the same is also true for RI-EOM-CCSD. Hence, the efficiency of our DF-EOM-CCSD implementation was maintained.

As the second step of our timing assessment, we consider the UHF versions of the RI-EOM-CCSD and DF-EOM-CCSD implementations. The DF-EOM-CCSD method noticeably reduces the computational cost compared with RI-EOM-CCSD (Figure 2); there is a 1.8-fold reduction in the computational time for DF-EOM-CCSD for the C_7_H_16_ molecule. For the C_7_H_16_ molecule, the CCSD time is 1168 and 920 min for RI-EOM-CCSD and DF-EOM-CCSD, respectively; there is a 1.3-fold reduction in the computational time for DF-EOM-CCSD. Further, for the C_7_H_16_ molecule, the EOM time is 3221 min (RI-EOM-CCSD) and 1568 min (DF-EOM-CCSD); hence, there is a 2.1-fold reduction in the computational time for DF-EOM-CCSD. The number of iterations for the UHF-CCSD part are 11 (DF-EOM-CCSD) and 12 (RI-EOM-CCSD) for the C_7_H_16_ molecule. The average computational time per UHF-CCSD iteration (*t*_*ccsd*_/*n*_*iter*_) for C_7_H_16_ is 83.7 min (DF-EOM-CCSD) and 97.3 min (RI-EOM-CCSD). Hence, our new DF-EOM-CCSD implementation is 1.2 times faster than the RI-EOM-CCSD code for the average computational cost per UHF-CCSD iteration. Similarly, the number of Davidson iterations for the EOM part are 12 (DF-EOM-CCSD) and 18 (RI-EOM-CCSD). The average computational time per Davidson iteration (*t*_*eom*_/*n*_*iter*_) for C_7_H_16_ is 130.7 min (DF-EOM-CCSD) and 178.9 min (RI-EOM-CCSD). Hence, our new DF-EOM-CCSD implementation is 1.4 times faster than the RI-EOM-CCSD code for the average computational cost per EOM-CCSD iteration. Hence, our new DF-EOM-CCSD implementation maintains its efficiency for the UHF reference.

#### 4.1.1 Assessment of the DF/CD Hybrid PPL Algorithm

**Figure 3:**
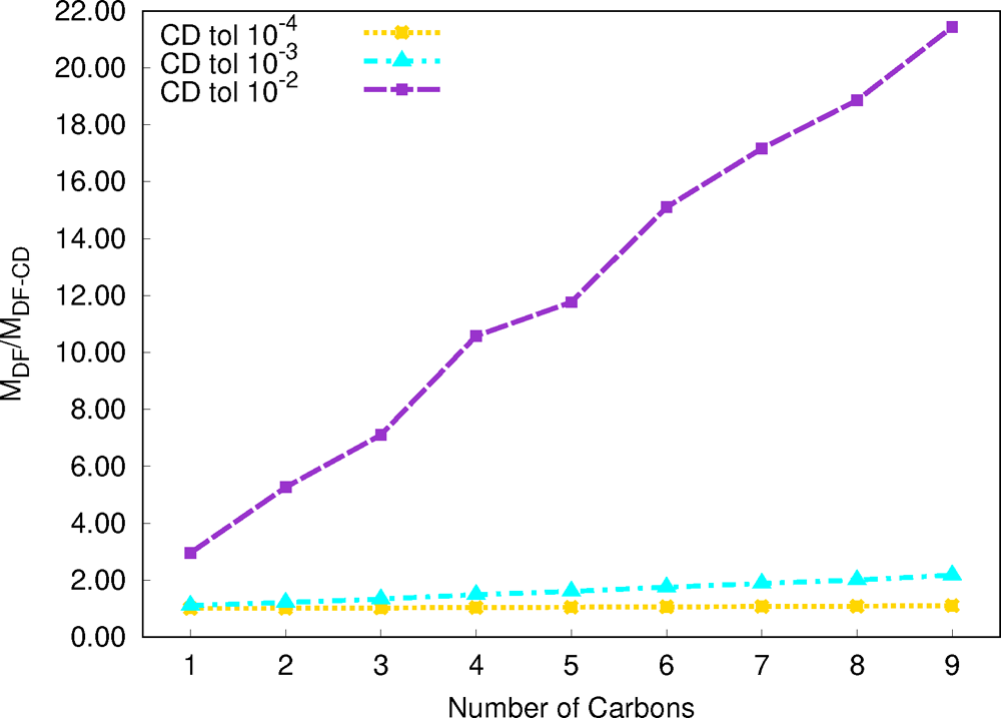
Ratio of the number of auxiliary basis functions, *M*, employed in the PPL term of DF-EOM-CCSD from the DF and hybrid DF/CD approaches (with the CD tolerances of 10^–4^, 10^–3^, and 10^–2^) for computations of excitation energies for the C_*n*_H_2*n*+2_ (*n* = 1–9) set. The RHF reference is used for these computations along with the cc-pVTZ basis set.

As the final step of our assessment for the efficiency of our new implementations, we present benchmark timing results for comparisons of DF and DF/CD hybrid approaches for the evaluation of the PPL terms of the CCSD and EOM parts. The ratios of the number of auxiliary basis functions employed in the PPL term of EOM-CCSD for the DF and DF/CD approaches are presented graphically in Figure 3. Since the most expensive term of the PPL algorithm scales linearly with the number of auxiliary basis functions, lets call it *M*, the reduction of *M* may yield significant improvements in the evaluation of the PPL term. For example, for the C_9_H_20_ molecule with the cc-pVTZ primary basis set, the *M* values are 1329 and 1208 for our canonical DF and hybrid DF/CD algorithms, respectively. Hence, the ratio of *M*_*DF*_/*M*_*DF*/*CD*_ is 1.10, which indicates a more than 10% reduction in the number of auxiliary basis functions. For the alkanes set considered, C_*n*_H_2*n*+2_ (*n* = 1–9), we plot the *M*_*DF*_/*M*_*DF*/*CD*_ values with respect to the *n* values and obtain a linear relation for this fit. The equation and the *R*^2^ value for the linear fit are *M*_*DF*_/*M*_*DF*/*CD*_ = 0.0122*n* + 0.9883 and *R*^2^ = 0.9939. At first, one should note that as the *n* value increases the *M*_*DF*_/*M*_*DF*/*CD*_ ratio increases as well. The reason for this correlation is that as molecular size increases the hybrid DF/CD algorithm makes better use of the sparsity of the ERI tensor. Hence, the obtained linear equation indicates that, if we proceed to larger molecules, the hybrid DF/CD algorithm will have a larger impact on the computational time. For example, the *M*_*DF*_/*M*_*DF*/*CD*_ ratio will be approximately 1.23 and 1.60 for the C_20_H_42_ and C_50_H_102_ molecules, which indicates up to 23% and 60% acceleration in the PPL terms can be achieved.

**Figure 4:**
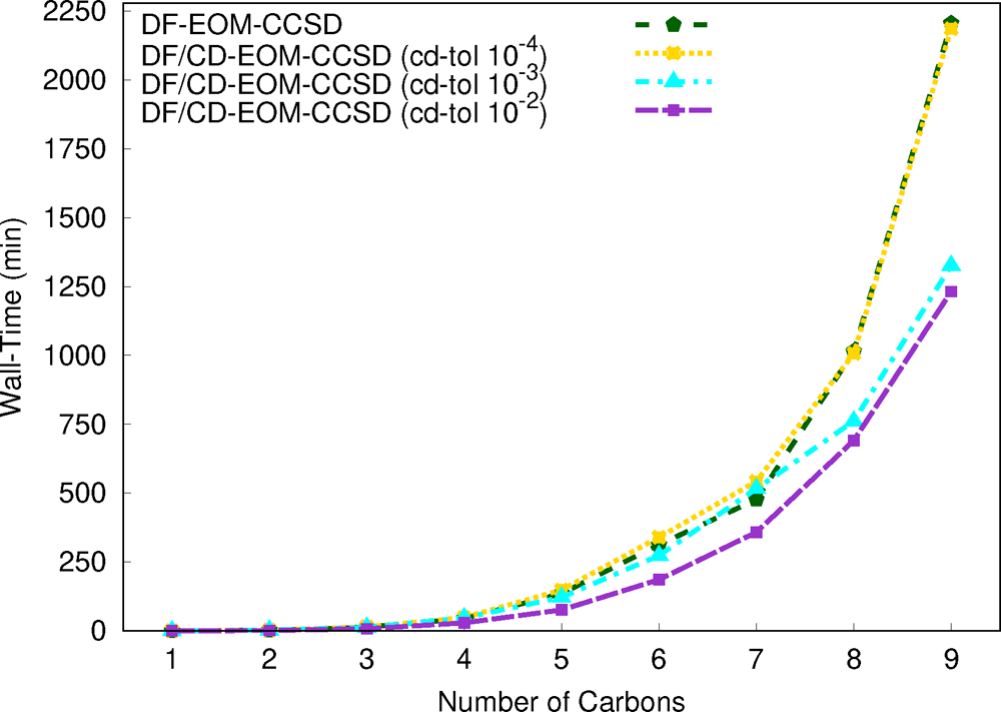
Total wall time (in min) for computations of excitation energies for the C_*n*_H_2*n*+2_ (*n* = 1–9) set from the DF-EOM-CCSD and hybrid DF/CD-EOM-CCSD (with the CD tolerances of 10^–4^, 10^–3^, and 10^–2^) methods with the cc-pVTZ basis set. The RHF reference is used for these computations. All computations were performed for a single root with 10^–7^ energy and EOM eigenvalue convergence tolerances on a single node (1 core) Intel(R) Xeon(R) CPU E5-2620 v4 @ 2.10 GHz computer (memory ∼ 64 GB).

For the alkanes set, the computational time for the DF-EOM-CCSD and hybrid DF/CD-EOM-CCSD approaches with the CD tolerances of 10^–4^, 10^–3^, and 10^–2^ are presented graphically in Figure 4. For the largest member of the test set, C_9_H_20_, the computational times are 2205.9 min (DF-EOM-CCSD), 2186.6 min (DF/CD-EOM-CCSD with *tol*_*CD*_ = 10^–4^), 1326.6 min (DF/CD-EOM-CCSD with *tol*_*CD*_ = 10^–3^), and 1232.0 min (DF/CD-EOM-CCSD with *tol*_*CD*_ = 10^–2^). With *tol*_*CD*_ = 10^–4^, the cost of DF/CD-EOM-CCSD is slightly reduced compared with that of DF-EOM-CCSD, while with *tol*_*CD*_ = 10^–3^ and *tol*_*CD*_ = 10^–2^, the cost of DF/CD-EOM-CCSD is reduced 39.9% and 44.1% compared with that of the canonical DF-EOM-CCSD. Hence, our new hybrid approach provides significant improvements in efficiency compared to the that of the canonical DF algorithm. Further, our above discussion suggests that for the larger molecules further improvements may be observed. Hence, our new hybrid DF/CD PPL algorithm appears to be very promising for large-sized chemical systems.

We would like to note why we did not prefer the CD decomposition of the original 4-index ERIs. The number of CD factors generated from the 4-index ERIs are generally much higher than that of the DF factors achieving the same accuracy. For example, for the C_8_H_18_ molecule with the cc-pVTZ basis set, the number of auxiliary basis functions are 1188 (DF), 2018 (*tol*_*CD*_ = 10^–4^), 1621 (*tol*_*CD*_ = 10^–3^), and 1005 (*tol*_*CD*_ = 10^–2^). Hence, the number auxiliary basis functions obtained from the partial CD decomposition of the conventional 4-index ERIs may yield a similar number with DF only if it is used with a loose CD tolerance of 10^–2^. However, with our hybrid approach, the number of auxiliary basis functions are 1095 (*tol*_*CD*_ = 10^–4^), 593 (*tol*_*CD*_ = 10^–3^), and 63 (*tol*_*CD*_ = 10^–2^). Hence, our hybrid DF/CD approach significantly reduces the number of auxiliary basis functions.

### 4.2 Accuracy of DF-EOM-CCSD

**Figure 5:**
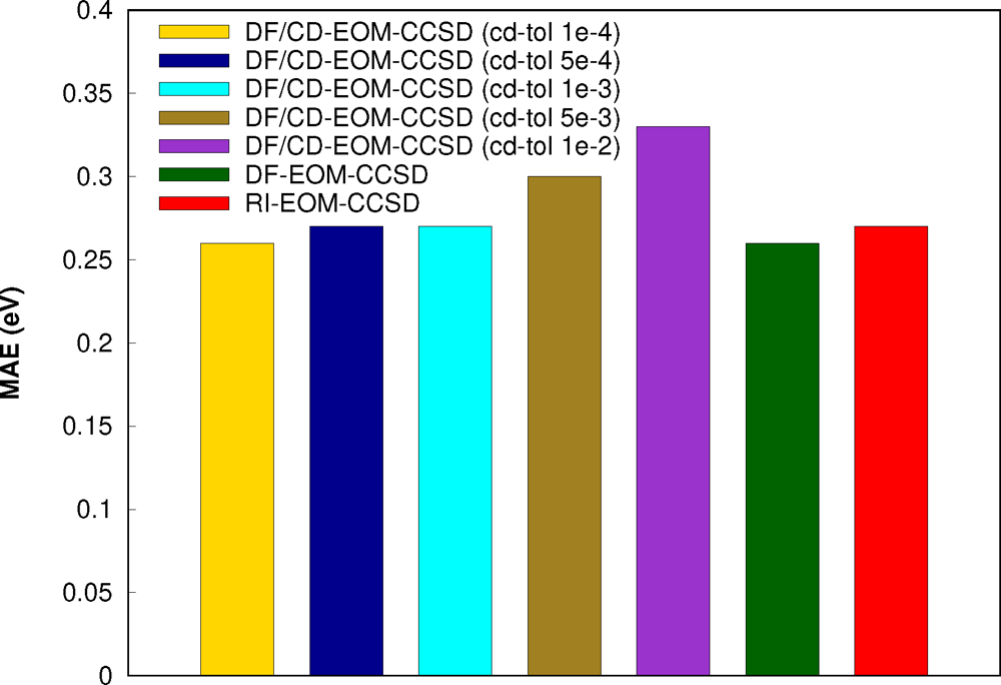
Mean absolute errors (in eV) in excitation energies for the test set from the DF-EOM-CCSD, DF/CD-EOM-CCSD, and RI-EOM-CCSD methods with respect to EOM-CCSD(fT) (the aug-cc-pVTZ basis set was employed).

In this section, we consider a test set to assess the accuracy of the DF-EOM-CCSD method. Chemical names of the molecules considered are given in the Supporting Information. Excitation energies (in eV) for the test set considered from the DF-EOM-CCSD, DF/CD-EOM-CCSD, RI-EOM-CCSD, and EOM-CCSD(fT) methods with the aug-cc-pVTZ basis set are reported in Table 1. The mean absolute errors (MAEs) with respect to EOM-CCSD(fT) are depicted in Figure 5. The MAE values with respect to EOM-CCSD(fT) are 0.71 eV (CIS), 0.26 eV (DF-EOM-CCSD), 0.26 eV (DF/CD-EOM-CCSD with *tol*_*CD*_ = 10^–4^), 0.27 eV (DF/CD-EOM-CCSD with *tol*_*CD*_ = 5 × 10^–4^), 0.27 eV (DF/CD-EOM-CCSD with *tol*_*CD*_ = 10^–3^), 0.30 eV (DF/CD-EOM-CCSD with *tol*_*CD*_ = 5 × 10^–3^), 0.33 eV (DF/CD-EOM-CCSD with *tol*_*CD*_ = 10^–2^), and 0.27 (RI-EOM-CCSD). Hence, the results of DF-EOM-CCSD, RI-EOM-CCSD, and DF/CD-EOM-CCSD (with *tol*_*CD*_ = 10^–4^–10^–3^) are almost identical. Further, the errors of DF/CD-EOM-CCSD with *tol*_*CD*_ = 5 × 10^–3^ and 10^–2^ are only 0.03 and 0.06 eV, deviating from the DF-EOM-CCSD approach. When these results and the computational efficiency are considered, the DF/CD-EOM-CCSD approach may be used with such loose CD tolerances. The differences between DF-EOM-CCSD and RI-EOM-CCSD results are in between 0.00 and 0.06 eV for most cases.

**Table 1:** Excitation Energies for the First Five Excited States (in eV) of the Test Set Considered from the DF-EOM-CCSD, DF/CD-EOM-CCSD, RI-EOM-CCSD, and EOM-CCSD(fT) Methods with the aug-cc-pVTZ Basis Set

	DF-EOM-CCSD	DF/CD-EOM-CCSD^a^	DF/CD-EOM-CCSD^b^	DF/CD-EOM-CCSD^c^	DF/CD-EOM-CCSD^d^	DF/CD-EOM-CCSD^e^	RI-EOM-CCSD^f^	EOM-CCSD(fT)^f^
1	5.78	5.78	5.78	5.78	5.78	5.79	5.83	5.51
	6.67	6.67	6.67	6.68	6.71	6.73	6.70	6.41
	6.72	6.72	6.72	6.73	6.75	6.78	6.72	6.47
	7.33	7.33	7.33	7.33	7.34	7.36	7.39	7.07
	7.69	7.69	7.70	7.70	7.73	7.76	7.73	7.48
2	4.53	4.53	4.53	4.53	4.53	4.54	4.57	4.25
	6.57	6.57	6.57	6.58	6.61	6.63	6.60	6.36
	7.55	7.55	7.55	7.56	7.59	7.62	7.58	7.36
	7.60	7.60	7.61	7.61	7.64	7.67	7.63	7.40
	7.68	7.68	7.68	7.69	7.72	7.75	7.71	7.50
3	5.69	5.69	5.70	5.70	5.75	5.78	5.70	5.30
	5.87	5.87	5.88	5.89	5.96	6.01	5.86	5.58
	6.49	6.49	6.50	6.50	6.58	6.64	6.47	6.21
	6.54	6.54	6.55	6.56	6.63	6.69	6.52	6.25
	6.65	6.65	6.65	6.66	6.74	6.79	6.63	6.36
4	6.79	6.79	6.79	6.79	6.81	6.84	6.80	6.50
	6.92	6.92	6.92	6.92	6.95	6.98	6.91	6.62
	7.03	7.03	7.04	7.04	7.09	7.13	7.02	6.80
	7.42	7.42	7.42	7.43	7.47	7.51	7.41	7.18
	7.45	7.45	7.45	7.46	7.50	7.54	7.43	7.21
5	6.36	6.36	6.36	6.37	6.42	6.47	6.39	6.02
	6.45	6.45	6.45	6.46	6.53	6.60	6.43	6.16
	6.76	6.76	6.76	6.77	6.84	6.91	6.74	6.47
	6.92	6.92	6.93	6.93	7.00	7.08	6.90	6.63
	7.21	7.21	7.22	7.22	7.28	7.34	7.18	6.69
6	7.48	7.48	7.49	7.49	7.51	7.54	7.46	7.23
	8.09	8.09	8.09	8.09	8.10	8.12	8.08	7.81
	8.13	8.13	8.13	8.13	8.16	8.19	8.11	7.89
	8.20	8.20	8.20	8.20	8.23	8.26	8.18	7.95
	8.55	8.55	8.55	8.56	8.57	8.59	8.56	8.36
7	4.07	4.07	4.07	4.07	4.07	4.07	4.07	3.82
	7.20	7.20	7.20	7.20	7.21	7.22	7.22	7.07
	8.09	8.09	8.09	8.09	8.09	8.10	8.11	7.97
	8.18	8.18	8.18	8.18	8.19	8.21	8.20	8.07
	8.61	8.61	8.61	8.61	8.62	8.63	8.64	8.52
8	5.70	5.70	5.70	5.70	5.70	5.70	5.74	5.45
	6.92	6.92	6.93	6.93	6.94	6.96	6.92	6.68
	6.99	6.99	7.00	7.00	7.01	7.03	7.01	6.71
	7.51	7.51	7.51	7.51	7.52	7.54	7.57	7.31
	7.71	7.71	7.71	7.72	7.73	7.74	7.76	7.53
9	6.19	6.19	6.20	6.20	6.27	6.32	6.18	5.88
	6.57	6.57	6.57	6.58	6.62	6.65	6.53	6.09
	6.73	6.73	6.74	6.74	6.81	6.86	6.72	6.43
	6.89	6.89	6.89	6.89	6.91	6.93	6.90	6.60
	6.92	6.92	6.92	6.93	7.00	7.05	7.34	7.03
10	5.82	5.82	5.83	5.84	5.89	5.93	5.81	5.50
	6.59	6.59	6.60	6.60	6.65	6.70	6.58	6.29
	6.63	6.63	6.63	6.63	6.67	6.70	6.59	6.16
	6.85	6.85	6.85	6.86	6.89	6.90	6.83	6.54
	6.87	6.87	6.87	6.88	6.91	6.96	6.91	6.49

a These computations were performed with the hybrid DF/CD algorithm employing a CD tolerance of 1 × 10^–4^.

b These computations were performed with the hybrid DF/CD algorithm employing a CD tolerance of 5 × 10^–4^.

c These computations were performed with the hybrid DF/CD algorithm employing a CD tolerance of 1 × 10^–3^.

d These computations were performed with the hybrid DF/CD algorithm employing a CD tolerance of 5 × 10^–3^.

e These computations were performed with the hybrid DF/CD algorithm employing a CD tolerance of 1 × 10^–2^.

f These computations were performed with the Q-chem 5.3 program.

## 5 Conclusions

In this study, a new implementation of the density-fitted EOM-CCSD (DF-EOM-CCSD) method has been presented with an enhanced algorithm for the particle–particle ladder (PPL) term, which is the most expensive term of the EOM-CCSD computations. To further improve the evaluation of the PPL term, a hybrid DF/CD algorithm has also been introduced. The computational time of the DF-EOM-CCSD excitation energies has been compared with that of the RI-EOM-CCSD method (from Q-chem 5.3 package^72^).

The DF-EOM-CCSD method significantly reduces the computational cost compared to that of the RI-EOM-CCSD method; there is more than a 2-fold reduction for the C_8_H_18_ molecule in a cc-pVTZ basis set with the RHF reference. This cost savings results from the accelerated evaluation of the PPL term. In our RHF based DF-EOM-CCSD method, the number of flops (NOFs) for the PPL contraction term is 2.5 times lower than that of the RI-EOM-CCSD method. Further, the prefactor of the *VVVV*-type transformation step used in the PPL term has a reduced NOF value by a factor of 2. For the UHF reference, our implementation maintains its better performance and provides a 1.8-fold reduction in the computational cost compared to that of the RI-EOM-CCSD method for the C_7_H_16_ molecule. Furthermore, our results show that the suggested hybrid DF/CD algorithm further improves the canonical DF algorithm, and the degree of improvement increases as the molecular size increases. The preliminary results indicate that the new hybrid DF/CD PPL algorithm is very promising for large-sized chemical systems.

Finally, the DF-EOM-CCSD and DF/CD-EOM-CCSD methods are applied to a test set to compare the excitation energies with those from the CIS, RI-EOM-CCSD, and EOM-CCSD(fT) methods. Our results demonstrate that the DF-EOM-CCSD and DF/CD-EOM-CCSD methods (with CD tolerances of 10^–4^–10^–3^) provide identical results with to those of the RI-EOM-CCSD method. Further, the DF/CD-EOM-CCSD approach yields tolerable errors (0.03 and 0.06 eV) compared with those of the DF-EOM-CCSD method for the test set considered with loose CD tolerances, such as 5 × 10^–3^ and 10^–2^.

## Appendix A

### DF-CCSD 3-Index Intermediates

1- and 3-index intermediates used for DF-CCSD are given as follows:^65,66^

52

53

54

55

56

57

58

59

60

61

62

where

63

and *b*_*rs*_^*Q*^ values are the molecular orbital (MO) DF factors, which are defined in our previous studies.^65−67^

#### *F* and Intermediates

Density-fitted *F* and  intermediates are^65,66^

64

65

66

67

68

where *f*_*pq*_ is the MO basis Fock matrix. We would like to note that in our previous studies^65,66^ the diagonal parts of the Fock matrix were removed from the definitions of the *F*_*mi*_ and *F*_*ae*_ intermediates since the diagonal Fock terms were moved into the *t*_*ij*_^*ab*^*D*_*ij*_^*ab*^ definition in the amplitude equation.

#### *W* Intermediates

*W* intermediates, with the DF approximation, are^65,66^

69

70

71

where *P*_±_ (*pq*) is defined by

72

and  acts to permute the indices *p* and *q*.

#### and Intermediates

The  and  intermediates are defined as follows:^65,66^

73

74

75

## Supporting Information Available

Optimized geometries of the species; excitation energies with the cc-pVTZ basis set for the test set; CIS excitation energies with the aug-cc-pVTZ basis set for the test set (PDF)
Spin-free RHF-DF-EOM-CCSD equations (PDF)
