# Supplementary material for: Efficient Implementation of Equation-of-Motion Coupled-Cluster Singles and Doubles Method with the Density-Fitting Approximation: An Enhanced Algorithm for the Particle–Particle Ladder Term
Source: J Chem Theory Comput. 2022 Feb 2;18(3):1489–500. doi: 10.1021/acs.jctc.1c01000 (PMC8908769; doi:10.1021/acs.jctc.1c01000)
Supplement: Supplementary file 1 — ct1c01000_si_001.pdf [file ct1c01000_si_001.pdf]

Supporting Information for

Efficient Implementation of Equation-of-Motion Coupled-Cluster Singles and Doubles Method with the Density-Fitting Approximation: An Enhanced Algorithm for the Particle-Particle Ladder Term

Aslı Ünal and Uğur Bozkaya*

Department of Chemistry, Hacettepe University, Ankara 06800, Turkey

E-mail: ugur.bozkaya@hacettepe.edu.tr

Set of Alkanes

CH_4 (at the B3LYP/6-311G level)**

0 1

C	0.00000000	0.00000000	0.00000000
H	0.62760000	0.62760000	0.62760000
H	0.62760000	-0.62760000	-0.62760000
H	-0.62760000	0.62760000	-0.62760000
H	-0.62760000	-0.62760000	0.62760000

C_2H_6 (at the B3LYP/6-311G* level, from CCCBDB, http://cccbdb.nist.gov/)

0 1

C	0.00000000	0.00000000	0.76800000
C	0.00000000	0.00000000	-0.76800000
H	-1.01920000	0.00000000	1.15730000
H	0.50960000	0.88260000	1.15730000
H	0.50960000	-0.88260000	1.15730000
H	1.01920000	0.00000000	-1.15730000
H	-0.50960000	-0.88260000	-1.15730000
H	-0.50960000	0.88260000	-1.15730000

C_3H_8 (at the B3LYP/6-311G* level, from CCCBDB, http://cccbdb.nist.gov/)

0 1

C	0.00000000	0.58630000	0.00000000
C	-1.26810000	-0.26260000	0.00000000
C	1.26810000	-0.26260000	0.00000000
H	0.00000000	1.24490000	0.87600000
H	-0.00030000	1.24530000	-0.87580000
H	-2.15760000	0.37420000	0.00000000
H	2.15760000	0.37430000	0.00000000
H	-1.32710000	-0.90140000	0.88000000
H	-1.32710000	-0.90140000	-0.88000000

H 1.32710000 -0.90140000 -0.88000000

H 1.32720000 -0.90140000 0.88000000

C_4H_{10} (at the B3LYP/6-311G* level, from CCCBDB, http://cccbdb.nist.gov/)

0 1

C 0.00000000 0.76630000 0.00000000

C 0.00000000 -0.76630000 0.00000000

C -1.40520000 1.37260000 0.00000000

C 1.40520000 -1.37260000 0.00000000

H 0.55470000 1.12710000 0.87540000

H 0.55470000 1.12710000 -0.87540000

H -0.55470000 -1.12710000 0.87540000

H -0.55470000 -1.12710000 -0.87540000

H -1.37150000 2.46560000 0.00000000

H 1.37150000 -2.46560000 0.00000000

H -1.97340000 1.06060000 -0.88210000

H -1.97340000 1.06060000 0.88210000

H 1.97340000 -1.06060000 -0.88210000

H 1.97340000 -1.06060000 0.88210000

C_5H_{12} (at the B3LYP/6-311G* level, from CCCBDB, http://cccbdb.nist.gov/)

0 1

C 0.00000000 0.00000000 0.31470000

C 0.00000000 1.28340000 -0.52250000

C 0.00000000 -1.28340000 -0.52250000

C 0.00000000 2.55860000 0.32380000

C 0.00000000 -2.55860000 0.32380000

H 0.87580000 0.00000000 0.97750000

H -0.87580000 0.00000000 0.97750000

H 0.87530000 1.28380000 -1.18400000

H -0.87530000 1.28380000 -1.18400000

H	-0.87530000	-1.28380000	-1.18400000
H	0.87530000	-1.28380000	-1.18400000
H	0.00000000	3.45620000	-0.30120000
H	0.00000000	-3.45620000	-0.30120000
H	-0.88210000	2.60740000	0.97010000
H	0.88210000	2.60740000	0.97010000
H	0.88210000	-2.60740000	0.97010000
H	-0.88210000	-2.60740000	0.97010000

C_6H_{14} (at the B3LYP/6-311G* level, from CCCBDB, http://cccbdb.nist.gov/)

0 1

C	1.39160000	2.91240000	0.00000000
C	-1.39160000	-2.91240000	0.00000000
C	0.00000000	0.76600000	0.00000000
C	0.00000000	-0.76600000	0.00000000
C	-1.40320000	-1.38200000	0.00000000
C	1.40320000	1.38200000	0.00000000
H	-2.40550000	-3.32270000	0.00000000
H	2.40550000	3.32270000	0.00000000
H	0.87690000	3.30630000	0.88210000
H	0.87690000	3.30630000	-0.88210000
H	-0.87690000	-3.30630000	-0.88210000
H	-0.87690000	-3.30630000	0.88210000
H	1.95740000	1.02100000	-0.87520000
H	1.95740000	1.02100000	0.87520000
H	-1.95740000	-1.02100000	-0.87520000
H	-1.95740000	-1.02100000	0.87520000
H	-0.55380000	1.12930000	0.87600000
H	-0.55380000	1.12930000	-0.87600000
H	0.55380000	-1.12930000	-0.87600000
H	0.55380000	-1.12930000	0.87600000

C_7H_{16} (at the B3LYP/6-311G* level, from CCCBDB, http://cccbdb.nist.gov/)

0 1

C	0.00000000	0.00000000	0.49470000
C	0.00000000	1.28290000	-0.34370000
C	0.00000000	-1.28290000	-0.34370000
C	0.00000000	2.56680000	0.49370000
C	0.00000000	-2.56680000	0.49370000
C	0.00000000	3.84240000	-0.35260000
C	0.00000000	-3.84240000	-0.35260000
H	-0.87660000	0.00000000	1.15560000
H	0.87660000	0.00000000	1.15560000
H	-0.87650000	1.28380000	-1.00480000
H	0.87650000	1.28380000	-1.00480000
H	0.87650000	-1.28380000	-1.00480000
H	-0.87650000	-1.28380000	-1.00480000
H	0.87590000	2.56620000	1.15390000
H	-0.87590000	2.56620000	1.15390000
H	-0.87590000	-2.56620000	1.15390000
H	0.87590000	-2.56620000	1.15390000
H	0.00000000	4.73890000	0.27370000
H	0.88270000	3.88890000	-0.99820000
H	-0.88270000	3.88890000	-0.99820000
H	0.00000000	-4.73890000	0.27370000
H	-0.88270000	-3.88890000	-0.99820000
H	0.88270000	-3.88890000	-0.99820000

C_8H_{18} (at the B3LYP/6-311G* level, from CCCBDB, http://cccbdb.nist.gov/)

0 1

C	0.00250000	0.76660000	0.00000000
C	-0.00250000	-0.76660000	0.00000000
C	-1.40050000	1.38380000	0.00000000
C	1.40050000	-1.38380000	0.00000000
C	-1.40050000	2.91680000	0.00000000
C	1.40050000	-2.91680000	0.00000000
C	-2.80670000	3.52270000	0.00000000
C	2.80670000	-3.52270000	0.00000000
H	0.55630000	1.12660000	0.87680000
H	0.55630000	1.12660000	-0.87680000
H	-0.55630000	-1.12660000	0.87680000
H	-0.55630000	-1.12660000	-0.87680000
H	-1.95470000	1.02370000	-0.87660000
H	-1.95470000	1.02370000	0.87660000
H	1.95470000	-1.02370000	-0.87660000
H	1.95470000	-1.02370000	0.87660000
H	-0.84750000	3.27710000	0.87610000
H	-0.84750000	3.27710000	-0.87610000
H	0.84750000	-3.27710000	0.87610000
H	0.84750000	-3.27710000	-0.87610000
H	-2.77190000	4.61580000	0.00000000
H	-3.37280000	3.20880000	-0.88270000
H	-3.37280000	3.20880000	0.88270000
H	2.77190000	-4.61580000	0.00000000
H	3.37280000	-3.20880000	-0.88270000
H	3.37280000	-3.20880000	0.88270000

C_9H_{20} (at the AM1 level)

0 1

C	-4.99698600	0.36383200	-0.00000200
C	-3.75024200	-0.48195100	0.00000700
C	-2.49951800	0.37096000	-0.00000400
C	-1.24980000	-0.48288200	0.00000300
C	0.00000100	0.37106700	-0.00000300
C	1.24979800	-0.48288200	0.00000100
C	2.49951700	0.37096000	0.00000000
C	3.75024100	-0.48195100	-0.00000100
C	4.99698800	0.36383200	0.00000000
H	-5.90627300	-0.28447500	0.00000600
H	-5.03037100	1.01776700	0.90489100
H	-5.03037200	1.01774600	-0.90491000
H	-3.74726000	-1.14877500	0.90264700
H	-3.74726100	-1.14879700	-0.90261600
H	-2.49998300	1.03734700	-0.90267900
H	-2.49998000	1.03736800	0.90265600
H	-1.24977400	-1.14930400	0.90270600
H	-1.24977400	-1.14931800	-0.90269000
H	0.00000200	1.03748300	-0.90270800
H	0.00000400	1.03749300	0.90269400
H	1.24976800	-1.14930800	0.90270200
H	1.24976900	-1.14931400	-0.90269500
H	2.49998100	1.03735800	-0.90266700
H	2.49998200	1.03735700	0.90266700
H	3.74725700	-1.14878800	0.90262900
H	3.74725700	-1.14878500	-0.90263400
H	5.90627500	-0.28447800	0.00000100
H	5.03037800	1.01775600	-0.90490000
H	5.03037700	1.01775600	0.90490100

$C_{10}H_{22}$ (at the AM1 level)

0 1

C	5.63024600	-0.28503500	0.00000300
C	4.36362000	0.53064100	-0.00000300
C	3.13369200	-0.35196300	0.00000000
C	1.86390600	0.47171100	-0.00000300
C	0.63492500	-0.41191100	-0.00000100
C	-0.63492600	0.41191400	0.00000000
C	-1.86390600	-0.47171000	-0.00000300
C	-3.13369100	0.35196300	0.00000100
C	-4.36362000	-0.53064300	-0.00000300
C	-5.63024600	0.28503300	0.00000700
H	6.52375000	0.38486600	-0.00000400
H	5.67929500	-0.93796700	0.90491200
H	5.67929300	-0.93798500	-0.90489200
H	4.34466500	1.19721600	0.90263200
H	4.34466800	1.19720800	-0.90264300
H	3.15011400	-1.01815800	-0.90267200
H	3.15011400	-1.01815300	0.90267500
H	1.84791900	1.13794800	0.90270000
H	1.84792000	1.13794400	-0.90270900
H	0.65088500	-1.07813500	-0.90271100
H	0.65088600	-1.07813400	0.90270900
H	-0.65088700	1.07813400	0.90271200
H	-0.65088700	1.07813900	-0.90270800
H	-1.84792200	-1.13794100	-0.90271000
H	-1.84792000	-1.13794800	0.90269900
H	-3.15011100	1.01815100	0.90267800
H	-3.15011100	1.01816000	-0.90266900
H	-4.34466700	-1.19720600	-0.90264700
H	-4.34466400	-1.19722200	0.90262800

H	-6.52375200	-0.38486600	0.00000000
H	-5.67929400	0.93796600	0.90491500
H	-5.67929500	0.93798500	-0.90488800

Benchmark Set

Geometries presented below are taken from [Kánnár, D. and Szalay, P. G. 2014. Benchmarking coupled cluster methods on valence singlet excited states. Journal of chemical theory and computation, 10(9), 3757-3765.] which are optimized at the MP2/6-31G* level.

1-Acetamide

0 1

C	1.360012888	-0.343164579	-0.004143703
C	-0.076339324	0.146077119	-0.007436705
N	-1.031873618	-0.827044572	-0.005588741
O	-0.356650354	1.328760140	-0.001120857
H	1.816451380	-0.071068715	0.947476849
H	1.459108811	-1.418247420	-0.149557637
H	1.904652553	0.178216915	-0.789171868
H	-1.997600530	-0.547478634	0.007182606
H	-0.807245551	-1.803863852	-0.011645532

2-Acetone

0 1

C	0.000000000	0.000000000	0.184533914
O	0.000000000	0.000000000	1.393539117
C	-1.288329519	-0.000007571	-0.611807513
C	1.288329519	0.000007571	-0.611807513
H	-2.140675074	-0.000283976	0.062182601
H	2.140675074	0.000283976	0.062182601
H	-1.332914603	0.877057882	-1.261382322

H	-1.332681316	-0.876734901	-1.261850843
H	1.332914603	-0.877057882	-1.261382322
H	1.332681316	0.876734901	-1.261850843

3-Cyclopentadiene

0 1

H	0.000000000	-0.874682970	-1.873919542
H	0.000000000	0.874682970	-1.873919542
H	-2.205179343	0.000000000	-0.606592909
H	2.205179343	0.000000000	-0.606592909
H	-1.344917974	0.000000000	1.877425720
H	1.344917974	0.000000000	1.877425720
C	-1.176513449	0.000000000	-0.280409978
C	1.176513449	0.000000000	-0.280409978
C	0.000000000	0.000000000	-1.213270251
C	-0.732776483	0.000000000	0.987347529
C	0.732776483	0.000000000	0.987347529

4-Cyclopropene

0 1

C	0.847340224	-0.000009393	0.000000000
C	-0.514215096	0.643264872	0.000000000
C	-0.514202490	-0.643309917	0.000000000
H	1.443168258	-0.000003717	-0.910559528
H	1.443168258	-0.000003717	0.910559528
H	-1.055507919	1.571124592	0.000000000
H	-1.055478458	-1.571179556	0.000000000

5-E-butadiene

0 1

C	0.606997950	-0.399031152	0.000000000

C	-0.606997950	0.399031152	0.000000000
C	1.840518939	0.108705872	0.000000000
C	-1.840518939	-0.108705872	0.000000000
H	0.474444760	-1.476783653	0.000000000
H	-0.474444760	1.476783653	0.000000000
H	2.716018491	-0.525335359	0.000000000
H	2.010289007	1.178431092	0.000000000
H	-2.716018491	0.525335359	0.000000000
H	-2.010289007	-1.178431092	0.000000000

6-Ethene

0 1

C	0.6620555023	0.0000000000	-0.0000000000
C	-0.6620555023	-0.0000000000	0.0000000000
H	1.2316711058	0.9206569263	-0.0000000000
H	1.2316711058	-0.9206569263	-0.0000000000
H	-1.2316711058	-0.9206569263	0.0000000000
H	-1.2316711058	0.9206569263	0.0000000000

7-Formaldehyde

0 1

O	0.000000000	0.000000000	0.671855230
C	0.000000000	0.000000000	-0.527135527
H	0.936935231	0.000000000	-1.116314101
H	-0.936935231	0.000000000	-1.116314101

8-Formamide

0 1

C	-0.160660619	-0.385608410	0.000000000
O	-1.193140976	0.243913417	0.000000000
N	1.082952570	0.157816246	0.000000000

H	-0.132754009	-1.490219234	0.000000000
H	1.184051082	1.159008392	0.000000000
H	1.904603813	-0.418502293	0.000000000

9-Furan

0 1

O	0.000000000	0.000000000	1.158015618
C	-1.092690521	0.000000000	0.346054700
C	1.092690521	0.000000000	0.346054700
C	-0.715883197	0.000000000	-0.954960662
C	0.715883197	0.000000000	-0.954960662
H	-2.046501773	0.000000000	0.841081023
H	2.046501773	0.000000000	0.841081023
H	-1.369066297	0.000000000	-1.810055370
H	1.369066297	0.000000000	-1.810055370

10-İmidazole

0 1

N	-0.745357151	-0.811965874	0.000000000
C	-0.992296549	0.527763583	0.000000000
C	0.621171243	-0.974426229	0.000000000
N	0.118241171	1.222457711	0.000000000
C	1.131467726	0.293670158	0.000000000
H	-1.430781807	-1.546457173	0.000000000
H	-1.991610617	0.930432795	0.000000000
H	1.083978333	-1.944480327	0.000000000
H	2.166227232	0.590875896	0.000000000

Table S1. Excitation energies for the first 5-excited states (in eV) for the test set considered from the CIS method with the aug-cc-pVTZ basis set.

Molecule	CIS/ATZ
**1-Acetamide**	6.81
	7.63
	8.50
	8.70
	8.73
**2-Acetone**	5.26
	8.25
	9.14
	9.21
	9.25
**3-Cyclopentadiene**	5.52
	5.73
	6.27
	6.42
	6.45
**4-Cyclopropene**	6.81
	6.92
	7.26
	7.37
	7.40
**5-E-butadiene**	6.21
	6.25
	6.55
	6.71
	7.39
**6-Ethene**	7.21
	7.80
	7.80
	7.97
	8.60
**7-Formaldehyde**	4.63
	8.54
	9.36
	9.51
	9.82
**8-Formamide**	6.57
	7.70
	8.55
	8.61
	8.91
**9-Furan**	6.03
	6.37
	6.53
	6.78
	7.16
**10-İmidazole**	5.83
	6.47
	6.66
	6.75
	7.31

Table S2. Excitation energies for the first 5-excited states (in eV) for the test set considered from the CIS, DF-EOM-CCSD, DF/CD- EOM-CCSD (with the CD tolerance of 1e−4), RI-EOM-CCSD and EOM-CCSD(fT) methods with the cc-pVTZ basis set.

Molecule	CIS	DF-EOM-CCSD	DF/CD-EOM-CCSD	RI-EOM-CCSD	EOM-CCSD(fT)
Acetamide	6.86	5.91	5.91	5.94	5.63
	8.45	7.57	7.57	7.56	7.30
	9.18	7.66	7.66	7.67	7.29
	9.66	7.95	7.95	8.04	7.69
	9.95	8.91	8.91	8.90	8.64
Acetone	5.26	4.57	4.57	4.59	4.29
	9.54	7.65	7.65	7.67	7.37
	9.96	9.20	9.20	9.22	8.88
	10.07	9.42	9.42	9.59	9.25
	10.66	9.58	9.58	9.60	9.31
Cyclopentadiene	5.63	5.82	5.82	5.83	5.42
	7.36	7.21	7.21	7.17	6.60
	8.16	7.26	7.26	7.24	6.93
	8.38	8.17	8.17	8.15	7.83
	8.57	8.23	8.23	8.20	7.91
Cyclopropene	7.12	6.95	6.95	6.95	6.66
	7.42	7.26	7.26	7.25	6.95
	8.22	8.20	8.20	8.20	7.92
	8.79	8.60	8.60	8.58	8.35
	8.96	8.78	8.78	8.76	8.50
E-butadiene	6.55	6.65	6.65	6.69	6.30
	7.93	7.69	7.69	7.65	6.91
	7.95	7.89	7.89	7.89	7.58
	8.32	7.98	7.98	7.95	7.65
	8.67	8.04	8.04	8.03	7.72
Ethene	8.20	8.53	8.53	8.53	8.26
	8.54	8.68	8.68	8.65	8.42
	9.09	8.68	8.68	8.68	8.49
	9.43	9.46	9.46	9.44	9.21
	9.71	9.69	9.69	9.67	9.42
Formaldehyde	4.65	4.13	4.13	4.13	3.88
	9.84	8.31	8.31	8.32	8.12
	9.99	9.49	9.49	9.47	9.18
	10.06	9.85	9.85	9.86	9.54
	11.49	10.25	10.25	10.28	10.14
Formamide	6.62	5.83	5.83	5.85	5.57
	8.48	7.73	7.73	7.74	7.49
	9.03	7.76	7.76	7.75	7.35
	9.52	8.55	8.55	8.66	8.35
	10.45	8.73	8.73	8.72	8.47
Furan	6.68	6.89	6.89	6.84	6.38
	7.79	7.01	7.01	6.99	6.46
	8.27	7.73	7.73	7.71	7.39
	8.34	8.39	8.39	8.37	8.06
	8.81	8.61	8.61	8.59	8.25
Imidazole	7.21	6.91	6.91	6.89	6.54
	7.25	6.94	6.94	6.90	6.41
	7.98	6.99	6.99	7.02	6.60
	8.08	7.34	7.34	7.33	6.87
	8.24	8.04	8.04	8.03	7.73
