# Supplementary material for: Efficient Implementation of Equation-of-Motion Coupled-Cluster Singles and Doubles Method with the Density-Fitting Approximation: An Enhanced Algorithm for the Particle–Particle Ladder Term
Source: J Chem Theory Comput. 2022 Feb 2;18(3):1489–500. doi: 10.1021/acs.jctc.1c01000 (PMC8908769; doi:10.1021/acs.jctc.1c01000)
Supplement: Supplementary file 2 — ct1c01000_si_002.pdf [file ct1c01000_si_002.pdf]

Supporting Information for: Efficient Implementation of Equation-of-Motion Coupled-Cluster Singles and Doubles Method with the Density-Fitting Approximation: An Enhanced Algorithm for the Particle-Particle Ladder Term

Aslı Ünal[†,‡] and Uğur Bozkaya[*,‡]

†*Hacettepe University, Graduate School of Science and Engineering, Ankara 06800, Turkey*

‡*Department of Chemistry, Hacettepe University, Ankara 06800, Turkey*

E-mail: ugur.bozkaya@hacettepe.edu.tr

1 Spin Free Equations of DF-EOM-CCSD Model

Density-fitted spin free equations of the EOM-CCSD method are presented below.

2 DF-EOM-CCSD Intermediates

2.1 3-index Intermediates

$$\tilde{r}_{ij}^{ab} = 2r_{ij}^{ab} - r_{ji}^{ab} \tag{1}$$

$$R_{ia}^{Q} = \sum_{m}^{occ}\sum_{e}^{vir}\left(2r_{im}^{ae} - r_{mi}^{ae}\right)b_{me}^{Q} \tag{2}$$

Or:

$$R_{ia}^{Q} = \sum_{m}^{occ}\sum_{e}^{vir}\tilde{r}_{im}^{ae}b_{me}^{Q} \tag{3}$$

$$r^{Q} = 2\sum_{m}^{occ}\sum_{e}^{vir}r_{m}^{e}b_{me}^{Q} \tag{4}$$

$$r_{ij}^{Q} = \sum_{e}^{vir}r_{i}^{e}b_{je}^{Q} \tag{5}$$

$$r_{ab}^{Q} = \sum_{m}^{vir} r_{m}^{a} b_{mb}^{Q} \tag{6}$$

$$r_{ia}^{Q} = \sum_{e}^{vir} r_{i}^{e} b_{ae}^{Q} \tag{7}$$

$$r_{ai}^{Q} = \sum_{m}^{vir} r_{m}^{a} b_{mi}^{Q} \tag{8}$$

$$\tilde{r}_{ia}^{Q} = \sum_{e}^{vir} r_{i}^{e} t_{ae}^{Q} \tag{9}$$

$$\tilde{r}_{ai}^{Q} = \sum_{m}^{vir} r_{m}^{a} t_{im}^{Q} \tag{10}$$

$$r_{ia}^{Q\prime} = \sum_{m}^{occ} r_{im}^{Q} t_{m}^{a} \tag{11}$$

2.2 4-Index Intermediates

$$\mathcal{V}_{ijmn} = \sum_{e,f}^{vir} r_{ij}^{ef} \langle mn|ef \rangle \tag{12}$$

$$\mathcal{V}_{ijam} = \sum_{e,f}^{vir} r_{ij}^{ef} \langle am|ef\rangle_{DF} \tag{13}$$

$$R_{mnij} = \sum_{Q}^{Naux} r_{im}^{Q} \{ b_{nj}^{Q} + t_{jn}^{Q} \} \tag{14}$$

$$R_{mbif} = \sum_{Q}^{Naux} r_{im}^{Q} b_{bf}^{Q} \tag{15}$$

$$R_{mbij} = \sum_{e}^{vir} r_{i}^{e} \mathcal{Z}_{mbej} \tag{16}$$

$$\widetilde{R}_{mbij} = \sum_{e}^{vir} r_{i}^{e} \mathcal{Z}_{mbje} \tag{17}$$

2.3 2-Index Intermediates

$$R_{im} = \sum_{e}^{vir} r_{i}^{e} \mathcal{F}_{me} \tag{18}$$

$$X_{ij} = \sum_{Q}^{N_{aux}} \left(t_{ij}^{Q} + b_{ij}^{Q}\right) r^{Q} + \sum_{Q}^{N_{aux}} \sum_{e}^{vir} \left(R_{ie}^{Q} - r_{ei}^{Q} - \tilde{r}_{ei}^{Q}\right) b_{je}^{Q} \tag{19}$$

$$X_{ab} = \sum_{Q}^{aux}\sum_{m}^{occ}\Big(R_{ma}^{Q} + r_{ma}^{Q} - \tilde{r}_{ma}^{Q}\Big)b_{mb}^{Q} + \sum_{Q}^{aux}\Big(t_{ab}^{Q} - b_{ab}^{Q}\Big)r^{Q} \tag{20}$$

3 σ_0 Amplitude Equation

$$\sigma_0 = 2\sum_{i}^{occ}\sum_{a}^{vir} r_i^a F_{ia} + \sum_{ij}^{occ}\sum_{ab}^{vir}\tilde{r}_{ij}^{ab}\langle ij|ab\rangle \tag{21}$$

4 σ_1 Amplitude Equation

The spin orbital equations of the σ_1 expressions are as follows.

$$\begin{aligned}
\sigma_i^a &= \sum_{e}^{vir} r_i^e \mathcal{F}_{ae} - \sum_{m}^{occ} r_m^a \mathcal{F}_{mi} \\
&+ \sum_{m}^{occ}\sum_{e}^{vir} r_m^e\big(2\mathcal{W}_{maei} - \mathcal{W}_{maie}\big) + \sum_{m}^{occ}\sum_{e}^{vir}\tilde{r}_{im}^{ae}F_{me} \\
&- \sum_{Q}^{N_{aux}}\sum_{m}^{occ}\big(t_{im}^{Q} + b_{im}^{Q}\big)R_{ma}^{Q} + \sum_{Q}^{aux}\sum_{e}^{vir}R_{ie}^{Q}\big(b_{ae}^{Q} - t_{ae}^{Q}\big)
\end{aligned} \tag{22}$$

5 σ_2 Amplitude Equation

The spin orbital equations of the σ_2 expressions are as follows.

$$
\begin{aligned}
\sigma_{ij}^{ab} \;=\;& \hat{P}_{+}(ia,jb)\Bigg\{\sum_{e}^{vir} r_{ij}^{eb}\mathcal{F}_{ae} \;-\; \sum_{m}^{occ} r_{mj}^{ab}\mathcal{F}_{mi} \;-\; \sum_{m}^{occ} r_{m}^{a}\mathcal{W}_{mbij} \\
&+\; \frac{1}{2}\sum_{m}^{occ}\sum_{e}^{vir}\tilde{r}_{im}^{ae}\big(2\mathcal{W}_{mbej} \;-\; \mathcal{W}_{mbje}\big) \;-\; \frac{1}{2}\sum_{m}^{occ}\sum_{e}^{vir} r_{mi}^{ae}\mathcal{W}_{mbje} \;-\; \sum_{m}^{occ}\sum_{e}^{vir} r_{mj}^{ae}\mathcal{W}_{mbie} \\
&-\; \sum_{m}^{occ} t_{mj}^{ab}R_{im} \;+\; \sum_{Q}^{aux} r_{ia}^{Q}\Big(b_{jb}^{Q} + t_{jb}^{Q} - \tilde{t}_{jb}^{Q}\Big) \;-\; \sum_{Q}^{aux} r_{ib}^{Q\prime} t_{ja}^{Q} \\
&+\; \frac{1}{2}\sum_{m}^{occ}\sum_{e}^{vir} u_{im}^{ae}\big(2R_{mbej} \;-\; R_{mbje}\big) \;-\; \frac{1}{2}\sum_{m}^{occ}\sum_{e}^{vir} t_{mi}^{ae}R_{mbje} \;-\; \sum_{m}^{occ}\sum_{e}^{vir} t_{mj}^{ae}R_{mbie} \\
&-\; \sum_{m}^{occ} t_{m}^{a}\big(R_{mbij} - \widetilde{R}_{mbji}\big) \;-\; \sum_{e}^{vir} X_{ae}t_{ij}^{eb} \;-\; \sum_{m}^{occ} X_{im}t_{mj}^{ab}\Bigg\} \\
&+\; \sum_{m,n}^{occ} \tau_{mn}^{ab}\big(R_{mnij} + R_{nmji} + \mathcal{V}_{ijmn}\big) \;+\; \sum_{e,f}^{vir} r_{ij}^{ef}W_{abef} \;+\; \sum_{m,n}^{occ} r_{mn}^{ab}\mathcal{W}_{mnij} \qquad (23)
\end{aligned}
$$
